# AMPs inhibit the proliferation, migration, and invasion of lung cancer via the CHRM3/PI3K/AKT and CHRM3/MAPK pathways

**DOI:** 10.3389/fonc.2025.1582040

**Published:** 2025-07-11

**Authors:** Jun Wu, Congcong Huang, Shangning Wang, Liuyan Chen, Quan Cheng, Shao Zhang

**Affiliations:** ^1^ Department of Thoracic Surgery, Hainan Cancer Hospital, The Affiliated Cancer Hospital of Hainan Medical University, Haikou, Hainan, China; ^2^ Department of Laboratory Medicine, Hainan Cancer Hospital, The Affiliated Cancer Hospital of Hainan Medical University, Haikou, Hainan, China; ^3^ Department of Medical Oncology, Hainan Cancer Hospital, The Affiliated Cancer Hospital of Hainan Medical University, Haikou, Hainan, China

**Keywords:** M3 muscarinic receptor, Armillaria mellea polysaccharide, migration, invasion, PI3K/AKT, MAPK, antitumor activity

## Abstract

**Aims:**

Our previous studies indicated that the overexpression of M3 muscarinic receptor (M3R/CHRM3) is related to a poor prognosis in patients with lung cancer and that *Armillaria mellea* polysaccharides (AMPs) can exhibit strong anticancer activity *in vitro* via apoptosis-related mechanisms in lung cancer cells. This study investigated whether AMPs exert anticancer activity through the CHRM3 signaling pathway.

**Materials and methods:**

Lung cancer cell lines (A549, NCI-H1299, and NCI-H520) with stable overexpression or knockdown of CHRM3 were established by infection with recombinant lentivirus and selected under puromycin for one month. Stable cells were treated with or without 100 μg/mL AMPs for 24 h or 48 h. The changes in CHRM3 expression, cell proliferation, migration, and invasion were determined. The expression levels of phosphoinositide 3-kinase (PI3K)/protein kinase B (AKT) and mitogen-activated protein kinase (MAPK) pathway-related proteins were detected. The antitumor activity of AMPs was further assessed in a xenograft mouse model bearing A549 cells with stable CHRM3 knockdown.

**Results:**

CHRM3 was highly expressed in NCI-H520 cells and moderately expressed in A549 and NCI-H1299 cells. CHRM3 overexpression significantly increased while CHRM3 knockdown significantly decreased the cell proliferation, migration, and invasion. AMP treatment downregulated the expression of CHRM3 and decreased the cell proliferation, migration, and invasion. Moreover, CHRM3 overexpression significantly activated the PI3K/AKT and MAPK signaling pathways, whereas AMP treatment decreased CHRM3-induced PI3K/AKT and MAPK activation. In xenograft mice bearing A549 tumors, CHRM3 knockdown showed little inhibition on tumor growth, but AMP treatment inhibited the tumor growth.

**Conclusion:**

AMP treatment inhibits the proliferation, migration, and invasion of lung cancer via the CHRM3/PI3K/AKT and CHRM3/MAPK pathways, thus exerting antitumor activity.

## 1 Introduction

Lung cancer is one of the most commonly diagnosed cancers, with an estimated 2.2 million new cases in 2020, accounting for 11.4% of all cancer cases worldwide. It is also the leading cause of cancer-related deaths worldwide, with 1.8 million deaths, accounting for 18.0% of cancer-related deaths in 2020 (1, 2). Lung cancer can be histopathologically classified into non-small-cell lung cancer (NSCLC) and small-cell lung carcinoma (SCLC). Approximately 85% of lung cancer patients have NSCLC, which is further histologically categorized as squamous cell carcinoma (SCC), adenocarcinoma, and large-cell carcinoma (3, 4). Nowadays, optional treatments include surgery, chemoradiotherapy, and targeted therapies (5). The advent of tyrosine kinase inhibitors and immune checkpoint inhibitors completely changed the therapeutic landscape of NSCLC and have provided considerable survival improvements in selected patients (4). However, drug resistance remains common, secondary resistance appears within two years for most patients, and most patients eventually experience disease progression during immunotherapy (5). Moreover, patients with NSCLC are often currently diagnosed with metastatic disease, together with drug resistance, resulting in a frustratingly low five-year overall survival of less than 30% (6–9).

The muscarinic receptors (MRs) are a group of five related receptors belonging to the G-protein coupled receptor family, with five subtypes (M1R–M5R) encoded by the cholinergic receptor muscarinic (CHRM) 1–5 genes (10, 11). MRs have been highly conserved throughout evolution and regulate a wide range of biological processes by modulating the epidermal growth factor receptor signaling pathway (10, 11). MRs are responsive to acetylcholine (ACh), a neurotransmitter that generally functions in the central and peripheral nervous systems and also plays an important role in tumorigenesis (12). Overexpression of MRs is associated with cell proliferation, apoptosis, angiogenesis, and even epithelial–mesenchymal transition, leading to cancer progression (13, 14). Evidence has shown that muscarinic agonists can stimulate tumor growth, while M3R antagonists inhibit tumor growth in breast, melanoma, lung, gastric, colon, pancreatic, ovarian, prostate, and brain cancer (12). Both our previous study (15) and another recent study (16) have suggested that the overexpression of M3R is related to a poor prognosis in patients with NSCLC. In NSCLC cells, activation of M3R can promote cell proliferation and invasion via the epidermal growth factor receptor (EGFR)/phosphoinositide 3-kinase (PI3K)/protein kinase B (AKT) pathway (17). Moreover, muscarinic antagonist treatment inhibited the PI3K/AKT and MEK/ERK1/2 pathways, thus inhibiting tumor growth (16).

PI3K is an intracellular phosphatidylinositide kinase. Activated PI3K can be recruited to the cell membrane, leading to the phosphorylation of phosphatidylinositol 4,5-bisphosphate and then phosphatidylinositol (3,4,5)-trisphosphate, thus promoting the phosphorylation of Akt. The PI3K/AKT signaling pathway has been reported to be involved in many cancers. Mitogen-activated protein kinases (MAPKs), a group of evolutionarily conserved serine/threonine protein kinases, can be divided into three main subfamilies, namely, extracellular signal regulated kinases (ERKs), Jun N-terminal kinases (JNKs), and the p38 MAPK. MAPKs are involved in various biological processes, including cell growth, apoptosis, hormone signaling, immune response, and inflammation, thus playing an important role in various cancers.

Natural bioactive compounds have shown great potential in the field of cancer immunotherapy due to their multi-target regulated abilities in recent years (18–20). Polysaccharides, one type of bioactive compounds, are well known for their antioxidation, immunomodulatory, antitumor, anti-inflammatory, and hypoglycemic activity (21, 22). Many types of polysaccharides are reported to exert antitumor activity (23). Of them, mushroom-derived polysaccharides and polysaccharide–protein complexes are considered as one of the major sources (24). *Armillaria mellea*, an edible and medicinal fungus, has been used for hundreds of years in East Asia. *A. mellea* polysaccharides (AMPs) have been reported to be multifunctional. They exhibit antioxidant activities by superoxide radical scavenging (25). AMPs can protect against L-Glu-induced neurotoxicity and mitigate Alzheimer’s disease-like behaviors by modulating oxidative stress (26).

Mushrooms have long been recognized as a source of bioactive compounds that interact with muscarinic receptors. Muscarine, the first muscarinic agonist ever isolated, was discovered in Amanita muscaria and shown to bind with high affinity to the M_3_ subtype (CHRM3), underscoring the ability of fungal metabolites to engage this receptor family. More recently, high-molecular-weight polysaccharides from various medicinal fungi have been characterized as “biological response modifiers,” capable of modulating receptor-mediated signaling in cancer cells. Although studies directly linking mushroom polysaccharides to downregulation of CHRM3 are scarce, classical PSP and lentinan preparations from Trametes versicolor and Lentinula edodes, respectively, inhibit PI3K/AKT and MAPK cascades—the same downstream effectors activated by CHRM3 in NSCLC—which provides a mechanistic rationale for hypothesizing that AMPs may similarly suppress CHRM3 expression and activity in lung cancer.

Our previous study indicated that AMPs can exhibit strong anticancer activity *in vitro* via apoptosis-related mechanisms in lung cancer cells (27). Moreover, our preliminary study showed that AMPs can significantly inhibit the expression of CHRM3. Therefore, we wondered if AMPs exert anticancer activity through the CHRM3 signaling pathway. This study aimed to investigate the underlying mechanism of AMPs against lung cancer.

## 2 Materials and methods

### 2.1 Cell culture, plasmid constructs, and lentivirus preparation

Six lung cancer cells NCI-H460 (large-cell carcinoma), NCI-H446 (small cell lung cancer), NCI-H520 (SCC), A549 (adenocarcinoma), NCI-H1299 (adenocarcinoma), and NCI-H1975 (adenocarcinoma), and human embryonic kidney cell line 293T were purchased from Puhao Bio. Ltd. (Nanjing, China). Cells were cultured in Dulbecco’s modified Eagle’s medium (DMEM; Gibco, USA) or RPMI 1640 (Perhor PH10014, China) with 10% fetal bovine serum (FBS; Gibco) and 100 mg/mL penicillin/streptomycin (Perhor PH10014, China).

The CHRM3-GFP gene sequence information was obtained from the National Center for Biotechnology Information gene database (reference sequence: NM_000740.4) and directly synthesized. The gene fragment was inserted into the XbaI-NotI sites of lentivirus overexpression vector Lenti-TK-PCDH-copGFP-T2A-Puro (Tsingke, Beijing, China) to construct the recombinant CHRM3-overexpression plasmid LV-CHRM3. Three shRNA-targeting CHRM3 sequences (sequence 1: 5′-GGACAGAGGCAGAGACAGA-3′; sequence 2: 5′-GGCAATACTTTGTTGGAAA-3′; sequence 3: 5′-TGGTGGACTTGGAGAGGAA-3′) were synthesized and inserted into the EcoRI-BamHI sites of lentivirus shRNA expression vector Lenti-U6-shRNA-GFP (Tsignke) to construct the recombinant CHRM3 shRNA plasmid LV-shCHRM. shRNA with a nontargeting sequence was used as a negative control. Recombinant plasmids were confirmed by sequencing.

Viral packaging was performed in 293T cells. Cells were cotransfected with recombinant plasmids, Lenti-GOI, and Lenti-packaging Mix (Tsingke) using LVTransm (iCARTAB, Jianshu, China). Viruses were collected at 48 h after transfection, and viral titers were assessed.

The lung cell lines A549, H1299, and H520 were infected with recombinant lentivirus-transducing units at a multiplicity of infection of 5. The expression of CHRM3 was detected using quantitative reverse transcription–polymerase chain reaction (qRT-PCR) and western blot.

The lung cell lines A549, H1299, and H520 cells stably infected with recombinant lentivirus were named A549-LV-CHRM3, A549-LV-NC, A549-LV-shCHRM3, and A549-LV-shNC; H1299-LV-CHRM3, H1299-LV-NC, H1299-LV-shCHRM3, and H1299-LV-shNC; H520-LV-CHRM3, H520-LV-NC, H520-LV-shCHRM3, and H520-LV-shNC; respectively. Lung cells with stable CHRM3 overexpression or knockdown were treated with or without 100 μg/mL AMPs (Bena, Henang, China) and then subjected to the assays.

Lung cancer cell lines with stable CHRM3 overexpression or knockdown and their controls are listed in **Table 1**. These cells were treated with or without 100 µg/mL AMPs (Bena, Henang, China) prior to all subsequent assays.

**Table 1:** Lentiviral constructs in lung cancer cell lines.

Cell Line	LV-CHRM3 (overexpression)	LV-NC (overexpression control)	LV-shCHRM3 (knockdown)	LV-shNC (knockdown control)
A549	A549-LV-CHRM3	A549-LV-NC	A549-LV-shCHRM3	A549-LV-shNC
H1299	H1299-LV-CHRM3	H1299-LV-NC	H1299-LV-shCHRM3	H1299-LV-shNC
H520	H520-LV-CHRM3	H520-LV-NC	H520-LV-shCHRM3	H520-LV-shNC

### 2.2 Cell counting kit-8 assay

Lung cells with stable CHRM3 overexpression or knockdown were seeded into 96-well plates at a concentration of 4×10^3^/mL and treated with or without 100 μg/mL AMPs. The CCK-8 assay was used to assess the proliferation of cells at 0, 24, 48, 72, and 96 h, according to the manufacturer’s instructions.

### 2.3 qRT-PCR

Total RNAs were extracted from the cells with TRIzol reagent (Invitrogen, USA), according to the manufacturer’s protocol. cDNA was first synthesized using a commercial kit (Thermo Fisher, USA). qPCR was performed with the Bio-rad DNA Engine Opticon 2 Real-Time PCR System in a 20-μL reaction volume using Real-time PCR Master Mix (SYBR Green) (Toyobo, Japan). The PCR amplification was performed as follows: 95°C for 3 s, followed by 40 cycles of 95°C for 10 s and 60°C for 20 s. The relative expression of target genes was analyzed using the 2^-△△CT^ method. GAPDH was used as an internal control. The primers used in the study were as follows: CHRM3: Sense primer: 5′-CACCATCCTCAACTCCACCAA-3′, Antisense primer: 5′-TCCATCGTCCACGCTCTTCT-3′; β-actin: Sense primer: 5′-CACCATGTACCCTGGCATTG-3′, Antisense primer: 5′-CCTGCTTGCTGATCCACATC-3′.

### 2.4 Western blot

Total protein was extracted from cells using RIPA lysis buffer with phosphatase inhibitors and proteinase inhibitors (Perhor). The protein concentration was determined using a bicinchoninic acid kit, according to the manufacturer’s instructions (Perhor). Proteins were first electrophoresed via sodium dodecyl sulfate–polyacrylamide gel electrophoresis and then transferred to polyvinylidene fluoride membranes. Membranes were blocked with 5% skim milk in TBS-T for 1 h at room temperature, then incubated overnight at 4°C with the primary antibodies listed in **Table 2**. Then, the membranes were incubated with horseradish peroxidase-labeled goat anti-mouse IgG secondary antibody (1:10000, Boster BA1050) or donkey anti-rabbit IgG secondary antibody (1:2000, Abcam ab6802). The bands were visualized using ECL substrate (Perhor) and quantified using the Focus523 system (Shenhua, Zhejiang, China). β-actin was used as an internal control.

**Table 2:** Primary antibodies used for Western blotting.

Target	Dilution	Supplier	Catalog No.
CHRM3	1:1000	Abclonal	A1602
AKT1	1:1000	Boster	BM1612
p-AKT1 (T450)	1:1000	Boster	BM4721
PI3Kp85	1:1000	Abclonal	A4992
p-PI3Kp85	1:1000	Abclonal	AP0057
p38	1:1000	Abclonal	A4771
p-p38 (Y182)	1:1000	Abclonal	AP0057
ERK1/2	1:1000	Abclonal	A4728
p-ERK1/2	1:1000	Abclonal	AP0974
JNK1/2/3	1:1000	Abclonal	A4867
p-JNK1/2/3	1:1000	Abclonal	AP0631
E-cadherin	1:1000	Boster	BM9561
β-actin	1:1000	Zen-Bio	200068-8F10

### 2.5 Scratch assay

The scratch assay was performed to determine the migration ability of stably infected lung cells treated with or without AMPs. Stably infected cells were seeded into six-well plates and were grown to 60% confluence; next, a linear wound was created in the cell monolayer with a pipette tip in each well. Detached cells were removed by washing the cell monolayer with phosphate-buffered saline (PBS). Cells were continually cultured with or without AMPs for 24 h. Images were captured under a light microscope at 100-fold magnification, and the migration distance was measured.

### 2.6 Transwell assay

The transwell assay was performed to evaluate the invasion ability of stably infected lung cells treated with or without AMPs. The assay was performed using Matrigel invasion chambers (Corning, USA). The transwell chambers were precoated with Matrigel. Stably infected cells were cultured in serum-free DMEM for 24 h and then added to the transwell chamber at a density of 1×10^5^/mL. DMEM containing 20% FBS was added to the lower chamber as the chemoattractant. Cells were cultured for 24 h, and noninvaded cells were removed. The remaining cells underneath the membrane surface were stained with 0.1% crystal violet at 37°C for 30 min. The cells were observed under a light microscope.

### 2.7 Immunofluorescence assay

Lung cells with stable CHRM3 overexpression or knockdown were seeded into six-well plates with pretreated coverslips for cells (Jinan, China). Cells were treated with AMPs for 24 h. After treatment, the cells were fixed with 4% paraformaldehyde in PBS at room temperature for 10 min. The cells were permeabilized with saponin (Beyotime, China) for 20 min and then blocked in PBS containing 5% bovine serum albumin for 40 min, followed by incubation with diluted primary antibody against CHRM3 (1:500, GeneTex GTX111637) overnight at 4°C. After being washed with PBS, the cells were incubated with DyLight 550-Conjugated AffiniPure Goat Anti-rabbit IgG (Boster BA1135, Wuhan, China) for 1 h in the dark, followed by 4′,6-diamidino-2-phenylindole staining for 5 min in the dark. The cells were finally imaged under a fluorescence microscope.

### 2.8 Tumor xenograft mouse model

All animal procedures were approved by the Ethics Committee of Hainan Cancer Hospital, The Affiliated Cancer Hospital of Hainan Medical University, and performed in accordance with the Guide for the Care and Use of Laboratory Animals. Twenty-four 4–5-week-old BALB/c nude mice (weighing 18–22 g) were purchased from Shanghai Lingchang Biotechnology Co., Ltd. The mice were maintained under controlled conditions (22–26°C, humidity of 50–60%, 12-h light/dark cycle) with access to food and water ad libitum.

Tumor cells (A549-LV-shNC and A549-LV-shCHRM3) were resuspended at a density of 1×10^7^/mL and were subcutaneously injected into the right axilla of the nude mice. Tumor growth was monitored by measuring the tumor size with digital calipers every three days. The greatest longitudinal diameter (length) and the greatest transverse diameter (width) were measured to determine the tumor volume as follows: 0.50×length×width^2^. Treatment was initiated when the average tumor volume reached 50 mm^3^. Mice bearing two tumor cells were randomly divided into four groups: A549-LV-shNC+saline, A549-LV-shNC+diamminedichloroplatinum (DDP) (5 mg/kg), A549-LV-shNC+low-dose AMPs (0.5 mg/kg), A549-LV-shNC+high-dose AMPs (2.5 mg/kg), A549-LV-shCHRM3+saline, A549-LV-shCHRM3+DDP, A549-LV-shCHRM3+low-dose AMPs, A549-LV-shCHRM3+high-dose AMPs (n=3 per group). A solution of AMPs or DDP was intravenously injected into mice once a week for three weeks. The appearance characteristics, physical activity, and water consumption of the mice were monitored every day. The body weight of each mouse was measured with an electronic scale every day. The mice were sacrificed by cervical dislocation after treatment for four weeks, and the tumors were dissected and weighed. The tumor volume was calculated.

### 2.9 Ethical compliance on maximal tumor size/burden in animal studies

(i) The Ethics Committee of Hainan Cancer Hospital (SEC-2019-018-01), The Affiliated Cancer Hospital of Hainan Medical University was no more than 20 mm in any dimension for any individual tumor.

(ii) The maximal tumor size/burden did not exceed the permitted limit during the study. All tumor sizes were regularly monitored, and no instances of exceeding the approved size were observed.

### 2.10 Statistical analysis

Data are shown as the mean±standard deviation from three independent experiments. Statistical analysis was performed using GraphPad Prism version 9.0.0 (GraphPad Software, San Diego, CA, USA). One-way analysis of variance with Tukey’s *post-hoc* analysis was used to compare the data in different groups. A p-value of less than 0.05 was considered statistically significant.

### 2.11 Dose selection and experimental replicates

To establish an effective *in vitro* working concentration, preliminary dose–response assays were performed in A549 cells treated with 25, 50, 100, and 200 µg/mL AMPs. A clear dose-dependent decrease in cell viability was observed, with an IC_50_ of approximately 100 µg/mL (data not shown). All *in vitro* experiments were conducted in three independent biological replicates, each comprising three technical replicates (n=3). For the xenograft study, each treatment arm included three mice (n=3 per group), and tumor measurements were taken in triplicate.

## 3 Result

### 3.1 Expression of CHRM3 in different lung cancer cell lines

**Figure 1:**
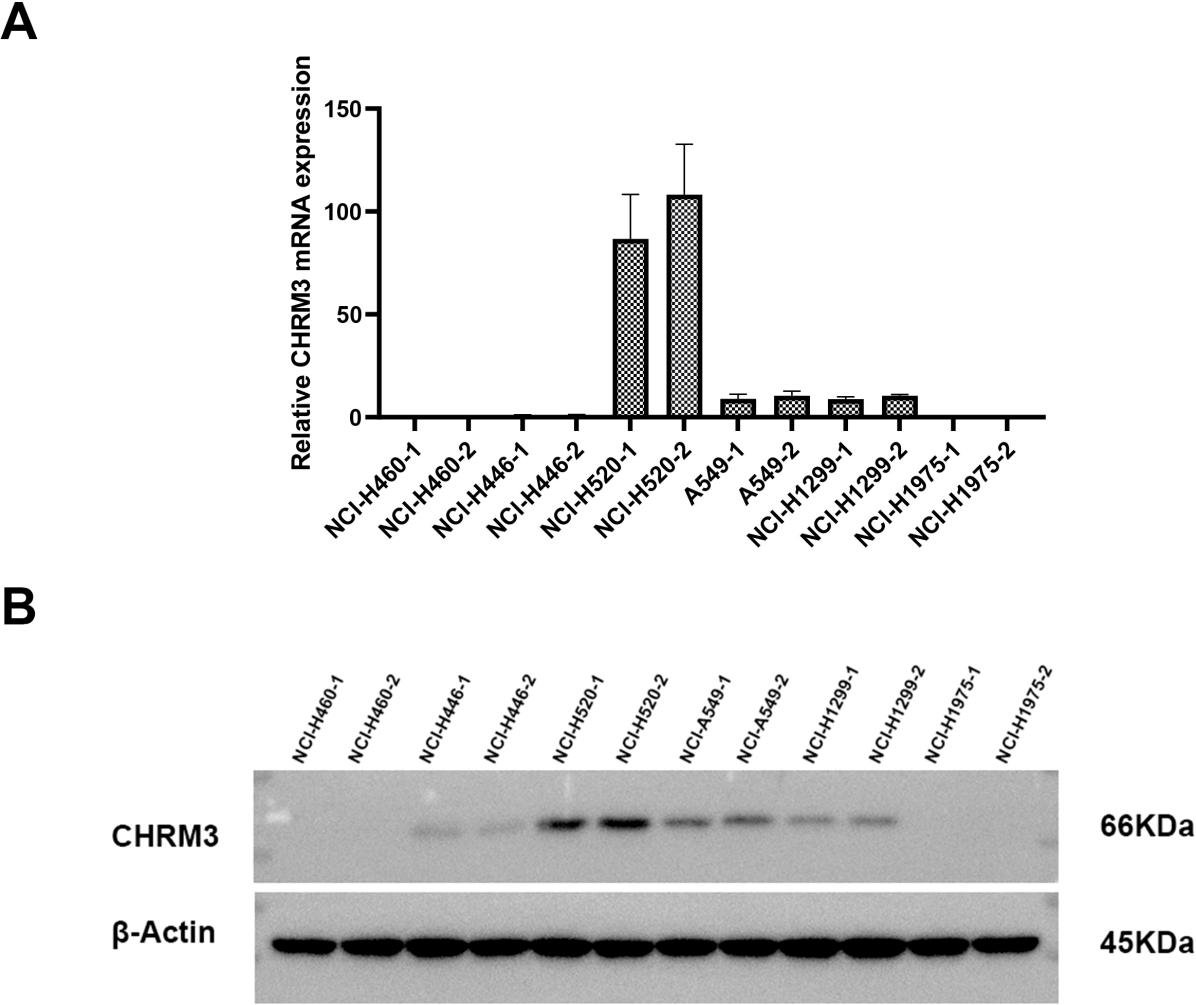
Expression of CHRM3 in six different lung cancer cell lines (NCI-H460, NCI-H446, NCI-H520, A549, NCI-H1299, and NCI-H1975). **(A)** qRT-PCR. **(B)** Western blot.

First, we assessed the expression of CHRM3 in six lung cancer cell lines (NCI-H460, NCI-H446, NCI-H520, A549, NCI-H1299, and NCI-H1975). Both the RT-qPCR and western blot data showed that CHRM3 was highly expressed in the SCLC-SCC NCI-H520 cell line, moderately expressed in the adenocarcinoma cell lines A549 and NCI-H1299, but not expressed in the other lung cancer cell lines (**Figure 1**). Therefore, the A549, NCI-H1299, and NCI-H520 cell lines were used for the following experiments.

### 3.2 Validation of overexpression and knockdown of CHRM3 in lung cancer cells

**Figure 2:**
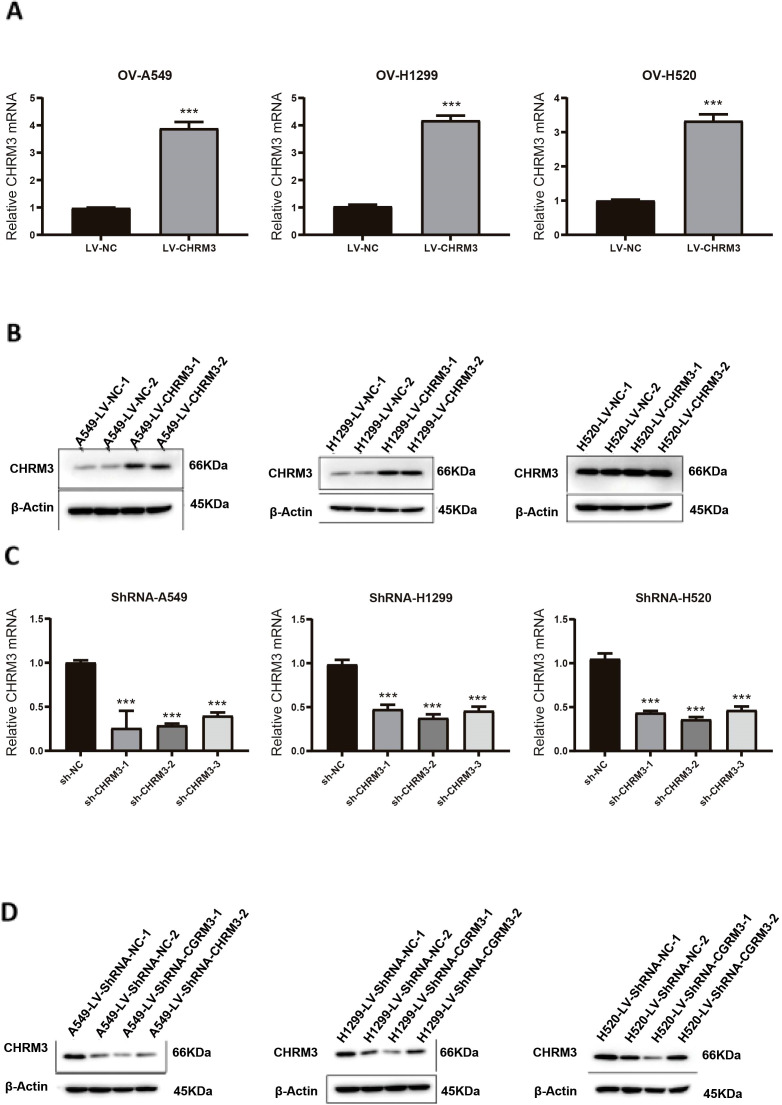
Validation of overexpression and knockdown of CHRM3 in three lung cell lines (A549, NCI-H1299, and NCI-H520) with baseline expression. Lung cells were infected with recombinant CHRM3 overexpression **(A, B)** or knockdown **(C, D)** lentivirus for 48 h. Cells were collected and the expression of CHRM3 was detected using qRT-PCR **(A, C)** or western blot **(B, D)**. *** *P* < 0.001.

Three lung cancer cell lines (A549, NCI-H1299, and NCI-H520) with baseline CHRM3 expression were infected with recombinant CHRM3-overexpression or -knockdown lentivirus. After infection, the expression of CHRM3 was determined using RT-qPCR and western blot assays. As shown in **Figures 2A, B**, our recombinant overexpression lentivirus significantly increased the expression of CHRM3 in the three lung cancer cell lines compared to the lentivirus control. **Figures 2C, D** shows that all three recombinant shRNA lentiviruses decreased the expression of CHRM3 in the three cell lines and that shRNA2 led to the lowest expression. Therefore, shRNA2 was used for the following experiments.

### 3.3 AMP treatment downregulated the expression of CHRM3 in lung cancer cells

**Figure 3:**
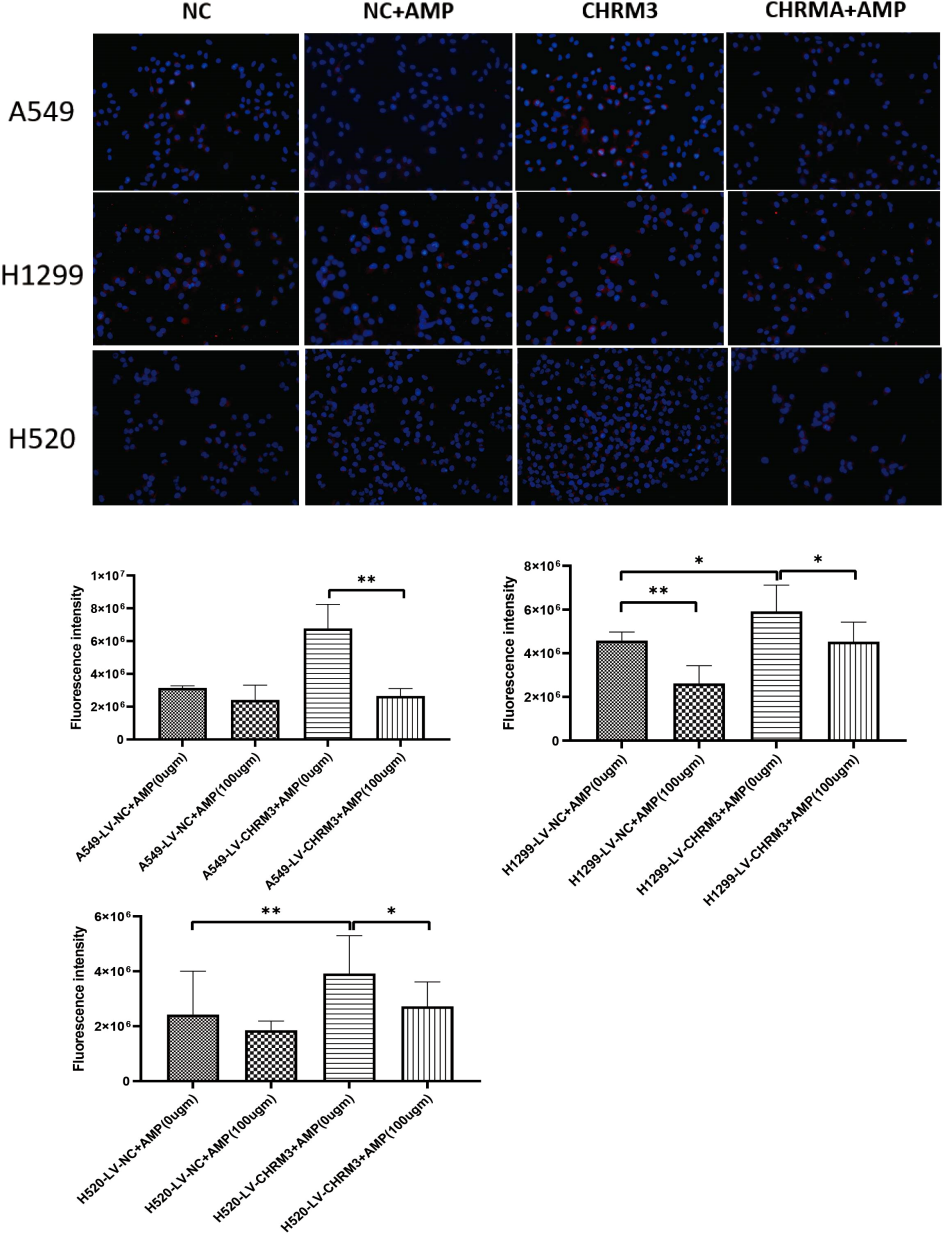
AMP treatment downregulates the expression of CHRM3. Lung cell lines (A549, NCI-H1299, and NCI-H520) with stable CHRM3 overexpression were established by infection with recombinant lentivirus and selected under puromycin for one month. Stable cells were treated with or without 100 μg/mL AMPs for 48 h. Expression of CHRM3 was detected using an immunofluorescence assay. * *P* < 0.05, ** *P* < 0.01.

Next, we established lung cancer cells (A549, NCI-H1299, and NCI-H520) with stable overexpression of CHRM3 by infection with recombinant CHRM3-overexpression lentivirus and cultured under puromycin for one month. Stable cells were treated with AMPs, and the effect of AMPs on the expression CHRM3 was detected. As shown in **Figure 3**, in the cells with stable NC, AMP treatment significantly decreased the expression of CHRM3 in the three cell lines. Moreover, AMP treatment decreased CHRM3 expression, especially in NCI-H1299 cells.

### 3.4 AMP treatment regulates lung cancer cell proliferation, migration, and invasion

**Figure 4:**
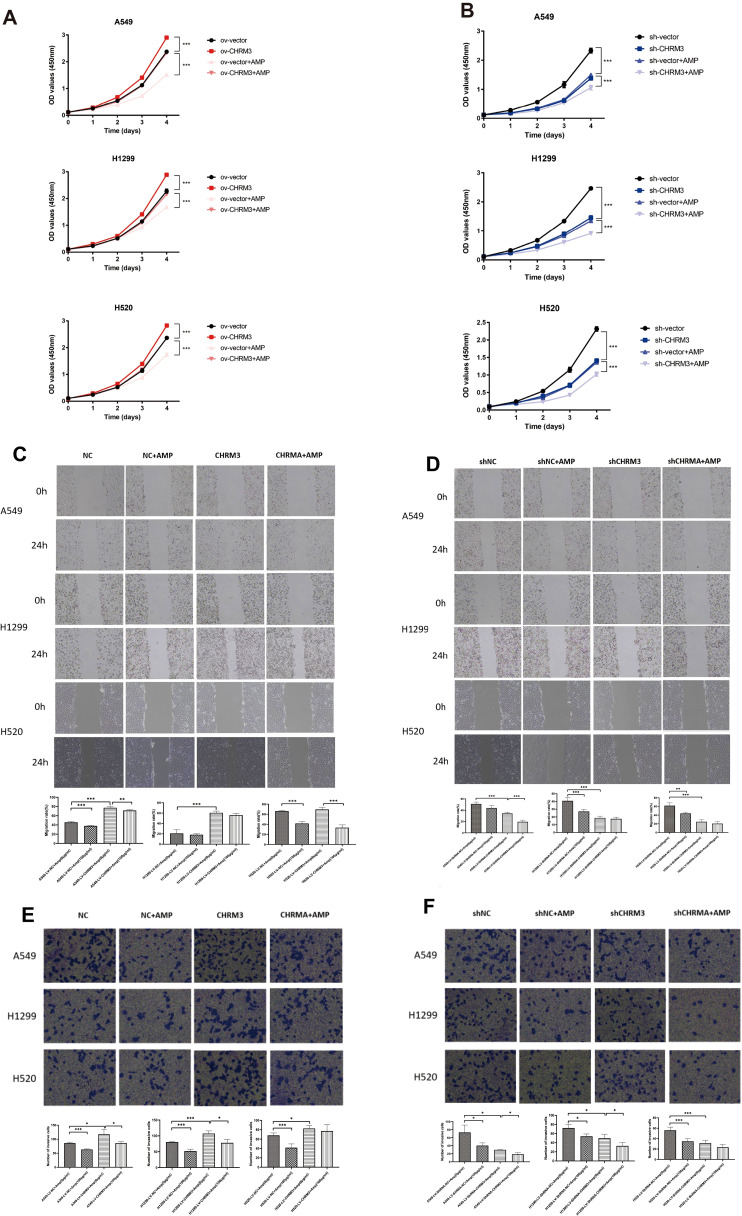
AMP treatment regulates lung cancer cell proliferation, migration, and invasion. Lung cells (A549, NCI-H1299, and NCI-H520) with stable CHRM3 overexpression **(A, C, E)** or knockdown **(B, D, F)** were treated with or without 100 μg/mL AMPs for 24 h. **(A, B)** The cell proliferation was detected using CCK-8 assays. **(C, D)** The cell migration ability was determined using a scratch assay. Images of the scratch were captured at 0 h and 24 h after the scratch was created. Representative images from three independent experiments are shown. **(E, F)** The cell invasion ability was determined using a transwell assay. * *P* < 0.05, ** *P* < 0.01, *** *P* < 0.001.

The effect of stable CHRM3 overexpression and knockdown on lung cell proliferation, migration, and invasion was first investigated and then whether AMP treatment affects them was also studied. As shown in **Figures 4A, B**, in all three cell lines, CHRM overexpression significantly increased cell proliferation, and AMP treatment significantly decreased CHRM overexpression-induced cell proliferation; while CHRM knockdown decreased cell proliferation, and AMP treatment further decreased cell proliferation.

**Figures 4C, D** shows the cell migration results. In A549 and NCI-H1299 cells, compared to the respective NC control cells, CHRM3 overexpression significantly increased cell migration, and AMP treatment significantly reversed this change in A549 cells but not in NCI-H1299 cells (*P*<0.01). AMP treatment also significantly decreased cell migration in CHRM3-overexpressed NCI-H520 cells (*P*<0.05) (**Figure 4C**). Compared to the respective shNC control cells, CHRM3 knockdown significantly decreased cell migration in A549 and NCI-H520 cells. AMP treatment significantly further decreased the cell migration in A549 cells (**Figure 4D**).

**Figures 4E, F** shows the cell invasion results. CHRM3 overexpression significantly increased cell invasion in all three cell lines, and AMP treatment significantly reversed this change in A549 and NCI-H1299 cells but not in NCI-H520 cells (**Figure 4E**). CHRM3 knockdown significantly decreased cell invasion in all three cell lines, whereas AMP treatment significantly decreased cell invasion in A549 and NCI-H1299 cells but not in NCI-H520 cells (**Figure 4F**).

### 3.5 AMP treatment regulates the PI3K/AKT and MAPK signaling pathways

**Figure 5:**
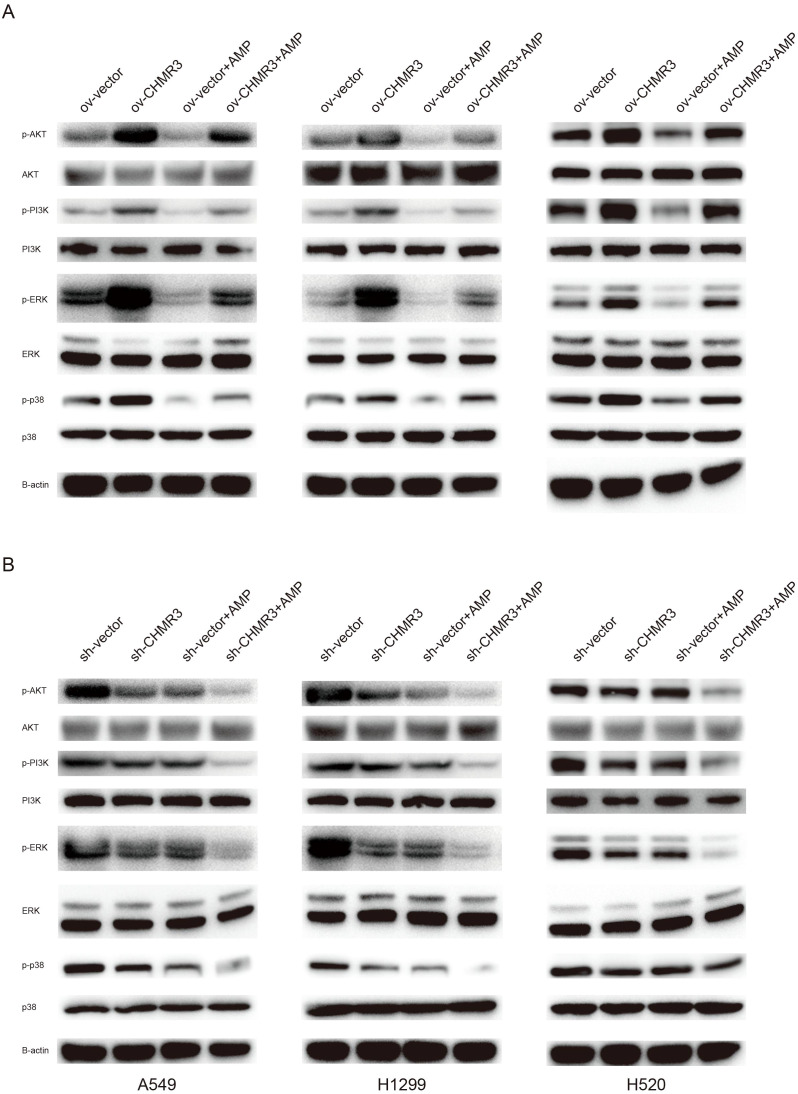
AMP treatment regulates the PI3K/AKT and MAPK signaling pathways. Lung cells (A549, NCI-H1299, and NCI-H520) with stable CHRM3 overexpression **(A)** or knockdown **(B)** were treated with or without 100 μg/mL AMPs for 48 h. The expression levels of PI3K/AKT pathway proteins and MAPK pathway proteins were detected using western blot.

As shown in **Figure 5**, compared to the respective shNC control cells, lung cells with stable CHRM3 overexpression significantly increased the phosphorylation protein levels of PI3K, AKT, P38, ERK, and JNK, while cells with stable CHRM3 knockdown significantly decreased the phosphorylation levels of PI3K, AKT, P38, ERK, and JNK proteins.

In cells with normal, overexpressed, or knockdown expressed CHRM3, AMP treatment significantly decreased the phosphorylation protein levels of PI3K, AKT, P38, ERK, and JNK. These data suggest that CHRM3 activated the PI3K/AKT and MAPK signaling pathways and that AMP treatment decreased CHRM3-induced PI3K/AKT and MAPK activation.

### 3.6 Antitumor activity of AMPs in the A549 xenograft mouse model

**Figure 6:**
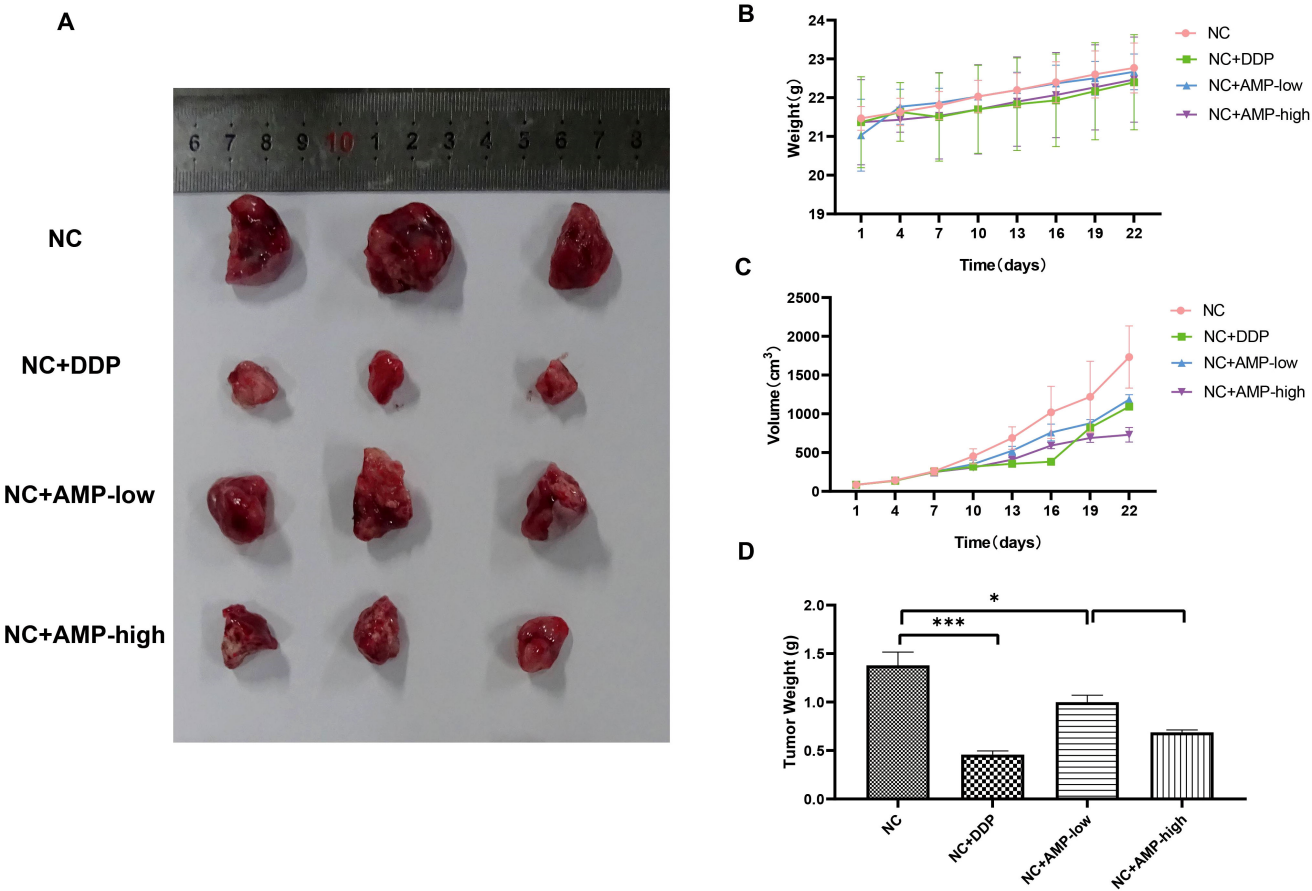
Antitumor activity of AMP treatment on a xenograft mouse model bearing A549 cells with stable CHRM3 knockdown. A549 cells were resuspended at a density of 1×10^7^/mL and were subcutaneously injected into the right axilla of the nude mice. Tumor growth was monitored by measuring the tumor size with digital calipers every three days. AMP treatment was initiated when the average tumor volume reached 50 mm^3^. Diamminedichloroplatinum (DDP) was used as a positive control. The body weight of each mouse was measured with an electronic scale every day. Mice were sacrificed after treatment for four weeks, and the tumors were dissected and weighed. **(A)** Tumor appearance. **(B)** Body weights of mice in eight different groups during the treatment. **(C)** Tumor volume in four different groups during the treatment. **(D)** The final tumor weights in four different groups. * *P* < 0.05, *** *P* < 0.001.

Next, we assessed the antitumor activity of AMPs in the A549 xenograft mouse model. The body weights of the mice in the different groups are shown in **Figure 6A**. There was no significant difference in the mouse body weight among all eight groups. The tumor growth curves of mice in the different groups are shown in **Figure 6B**, and the final tumor weights in the different groups are shown in **Figures 6C, D**. Both panels show that AMP treatment inhibited the tumor growth in a dose-dependent manner, and the inhibition in the high-dose AMP group was comparable to that of DDP.

## 4 Discussion

In the present study, we found that CHRM3 was highly expressed in the SCLC-SCC cell line NCI-H520 and moderately expressed in the adenocarcinoma cell lines A549 and NCI-H1299. We confirmed that CHRM3 overexpression significantly increased while CHRM3 knockdown significantly decreased cell proliferation, migration, and invasion. In addition, AMP treatment downregulated the expression of CHRM3 and decreased cell proliferation, migration, and invasion. Moreover, CHRM3 overexpression significantly activated the PI3K/AKT and MAPK signaling pathways, while AMP treatment decreased the activation of PI3K/AKT and MAPK that was upregulated by CHRM3. In xenograft mice bearing a A549 tumor, AMP treatment inhibited the tumor growth.

Besides being the principal neurotransmitter in the central and peripheral nervous systems, ACh is also synthesized and released by various non-neuronal cells (28). ACh in the airway epithelium is believed to regulate cell proliferation and thus is related to tumor progression (12). SCLC and SCC cells synthesize and release ACh, acting as an autocrine growth factor via both M3R and nicotinic ACh receptors (28). Consistent with this, here we found that CHRM3 was highly expressed in the SCLC-SCC cell line NCI-H520 and moderately expressed in the adenocarcinoma cell lines A549 and NCI-H1299. We previously found that a high CHRM3 expression was correlated with a poor survival in NSCLC patients (15). Here, we further observed that CHRM3 overexpression significantly increased cell proliferation, migration, and invasion. It has been reported that activation of CHRM3 by ACh promotes the proliferation, invasion, and migration of NSCLC cells via the EGFR/PI3K/AKT pathway (17). Another previous study has reported that blocking of M3R can inhibit lung cancer cell proliferation by limiting the activation of MAPKs (29). Furthermore, Lan et al. have reported that M3R antagonist treatment and knock out of M3R inhibited cell proliferation and migration as well as reduced the phosphorylation of EGFR. Moreover, the PI3K/AKT and MEK/ERK1/2 signaling pathways have been demonstrated to be involved in M3R-induced EGFR transactivation in NSCLC (16). In the present study, we found that CHRM3 overexpression significantly increased the phosphorylation levels of PI3K, AKT, P38, ERK, and JNK proteins, indicating activation of the PI3K/AKT and MAPK signaling pathways. Our results were consistent with previous reports.

*A. mellea* is an edible mushroom that has been traditionally used as an alternative medicine because of its antimicrobial and anticancer effects. *A. mellea* has shown anti-inflammatory activity by blocking the lipopolysaccharide-induced nitric oxide release and prostaglandin production as well as inducible nitric oxide synthase and cyclooxygenase-2 expression in the human monocytic cell line THP-1 (30). *A. mellea* can induce the maturation of human dendritic cells through a unique mechanism without inducing the production of tumor necrosis factor (TNF)-alpha, interleukin (IL)-12p40, or IL-10 (31). Moreover, *A. mellea* induces the expression of intercellular adhesion molecule-1 in THP-1 cells. It also has been shown to increase the phosphorylation of JNK and to increase the DNA-binding activity of the transcription factors nuclear factor-kappaB, activator protein-1, speckled protein-1, and signal transducer and activator of transcription-1 (32). Furthermore, Chang et al. purified xylosyl galactofucan from *A. mellea* and found that it can significantly suppress the release of TNF-alpha and cytokine monocyte chemotactic protein-1 in monocyte cells (33). Here, we observed that AMP treatment downregulated the expression of CHRM3. Previous studies have demonstrated that M3R antagonists can decrease the proliferative features and cell growth *in vitro* (29) and *in vivo* (34) in lung cancer. We also found that AMP treatment significantly inhibited lung cancer cell proliferation, migration, and invasion via downregulating CHRM3. Our data confirmed the antitumor activity of AMP treatment and broadened its possible clinical application. Moreover, further study indicated that AMPs can further inhibit the activation of PI3K, AKT, P38, ERK, and JNK proteins, which may also be through modulating CHRM3. This mechanism was to some extent consistent with Kim’s study (32), which demonstrated that *A. mellea* extract can activate the JNK signaling pathway.

Our results demonstrate that AMPs inhibit NSCLC cell proliferation, migration, and invasion in part by downregulating CHRM3 and attenuating PI3K/AKT and MAPK signaling. However, AMPs are well-known multifunctional bioactive polysaccharides, and their antitumor efficacy likely derives from a constellation of mechanisms. For example, AMPs can induce mitochondrial-mediated apoptosis in cancer cells, enhance antitumor immunity by promoting dendritic cell maturation and cytokine secretion, and fungal polysaccharides have been reported to inhibit angiogenesis via downregulation of VEGF and related pathways. Thus, while CHRM3 blockade contributes to the observed effects, the overall anticancer activity of AMPs is likely a synergistic result of CHRM3 inhibition together with apoptosis induction, immunomodulation, anti-angiogenesis, and other yet-to-be-elucidated pathways. Future work should dissect these parallel mechanisms through transcriptomic profiling, synergy assays with standard therapies, and orthotopic or patient-derived xenograft models, to fully understand and optimize the therapeutic potential of AMPs. Moreover, future studies should formally assess synergy between AMPs and current standard-of-care regimens—such as EGFR-targeted TKIs, platinum-based chemotherapies, and immune checkpoint inhibitors—using combination index analyses to identify additive or synergistic interactions. In parallel, direct evaluation of additional molecular pathways (e.g., quantification of apoptotic markers like cleaved caspase-3 and PARP, autophagy flux via LC3 conversion, angiogenesis regulators such as VEGF and CD31, and profiling of immune-modulatory cytokines) through targeted biochemical assays and unbiased omics approaches will further elucidate the full spectrum of AMP action.

From a translational standpoint, AMPs could be developed as an adjunct to existing regimens. For instance, co-administration with EGFR-targeted TKIs or immune checkpoint inhibitors may yield additive or synergistic tumor control by simultaneously targeting CHRM3-driven proliferation and enhancing apoptotic or immune-mediated clearance. Future work should evaluate pharmacokinetics, optimal dosing schedules and combination indexes in orthotopic and patient-derived xenograft models to inform the design of early-phase clinical trials.

Our animal study further confirmed that AMP treatment inhibits tumor growth, suggesting the potential therapeutic application of AMPs in lung cancer.

### 4.1 Limitations

Despite promising findings, this study has several limitations. *In vitro* experiments were limited to three NSCLC cell lines at a single AMP dose (100µg/mL), and *in vivo* testing used only a subcutaneous A549 xenograft at two doses with small group sizes (n=3), which may not capture full dose–response relationships or tumor heterogeneity. More clinically relevant models—such as orthotopic implantation and patient-derived xenografts—are needed to evaluate antitumor efficacy, biodistribution, pharmacokinetics, and pharmacodynamics. We did not perform formal synergy assays with standard therapies, nor investigate the upstream mechanisms by which AMPs downregulate CHRM3 (e.g., transcriptional or post-translational regulation). Additionally, comprehensive safety profiling, including toxicity and off-target effects in normal tissues, and validation in clinical samples are necessary. Addressing these gaps will require expanded preclinical studies, combination and synergy testing, and ultimately clinical trials to establish safety, optimal regimens, and therapeutic potential. Furthermore, our *in vivo* xenograft study was conducted with only n = 3 mice per group, which limits the statistical power and may increase result variability. Although we observed a consistent, dose-dependent inhibition of tumor growth across all three animals, future studies with larger cohort sizes are necessary to confirm the robustness and reproducibility of these findings.

## 5 Conclusion

Here, we found that AMP treatment inhibits the proliferation, migration, and invasion of lung cancer via the CHRM3/PI3K/AKT and CHRM3/MAPK pathways, thus exerting antitumor activity. AMPs might be a potential therapeutic alternative for lung cancer, especially for those who have failed first-line and second-line therapies.

## Data Availability

The original contributions presented in the study are included in the article/supplementary material. Further inquiries can be directed to the corresponding author.
